# Visualize neuronal membrane cholesterol with split-fluorescent protein tagged YDQA sensor

**DOI:** 10.1016/j.jlr.2025.100781

**Published:** 2025-03-19

**Authors:** Yi Xu, Saixuan Li, Yiran Xu, Xiaoqin Sun, Yuqing Wei, Yuejun Wang, Shuang Li, Yongqi Ji, Keyi Hu, Yuxia Xu, Cuiqing Zhu, Bin Lu, Dandan Wang

**Affiliations:** 1 State Key Laboratory of Medical Neurobiology and MOE Frontiers Center for Brain Science, Institutes of Brain Science, Fudan University, Shanghai, PR China; 2 Department of Endocrinology, Huadong Hospital Affiliated to Fudan University, Shanghai, PR China; 3 Department of Neurosurgery, Huashan Hospital, Fudan University, Shanghai, PR China

**Keywords:** Alzheimer’s disease, cholesterol, plasma membrane, sensor

## Abstract

Cholesterol is a major component of the cellular plasma membrane (PM), and its homeostasis is essential for brain health. Dysregulated cholesterol homeostasis has been strongly implicated in the pathogenesis of various neurological disorders, including Alzheimer’s disease (AD). However, in vivo visualization of cholesterol has remained challenging, hindering a comprehensive understanding of AD pathology. In this study, we generated a new sensor combining the split-fluorescent protein tags with YDQA, a derivate of cholesterol-dependent cytolysin PFO. Through a series of validations in cell and *C. elegans* models, we demonstrate that the new sensor (name as sfPMcho) efficiently detects neuronal PM cholesterol. We further applied this sensor in 5X FAD and APOE KO mice models and revealed the cholesterol changes within neurons. PM cholesterol became sparse and locally aggregated in neuron bodies but significantly accumulated in nerve fibers. Collectively, this study provides a new tool for detecting neuronal PM cholesterol in vivo and uncovers cholesterol abnormalities in AD-related pathology at the cellular level. Further development based on this sensor or a similar strategy is to be expected.

Cholesterol is a major component of the cellular plasma membrane (PM) and plays a crucial role in various physiological signaling processes (1). The brain contains approximately 20% of the body's total cholesterol despite comprising only about 2% of body weight (2). Unlike other tissues, the brain relies on its own cholesterol synthesis due to the impermeability of the blood-brain barrier (BBB) to circulating cholesterol. The majority of cholesterol in the brain is synthesized by astrocytes and transported by lipoproteins, primarily through apolipoprotein E (ApoE), which facilitates the delivery of cholesterol to neurons (3). To maintain homeostasis, excess cholesterol in neurons is converted into various oxysterols, such as 24S-hydroxycholesterol (24-OHC), by the enzyme CYP46A1 (4). Approximately 70%–80% of brain cholesterol resides in myelin, with the remaining part found in the PMs and membranous organelles of neurons and glia (5). Consequently, cholesterol participates in many aspects of synaptic function and cellular communication (6).

Maintaining cholesterol homeostasis is essential for brain health. Dysregulated cholesterol homeostasis is increasingly recognized as a contributing factor in the pathogenesis of neurodegenerative diseases, such as Alzheimer’s disease (AD) (7). AD is a leading cause of dementia in the elderly, in which patients suffer from memory loss and cognitive decline. There are several lines of evidence link cholesterol abnormalities to AD: 1) Cholesterol depletion in the neuronal PMs impairs neuronal activity (8, 9, 10); 2) Cholesterol affects the cleavage of amyloid precursor protein (APP) and the formation of amyloid plaques (11, 12, 13); 3) Cholesterol promotes tau aggregation (14, 15, 16); 4) ApoE4, a major risk factor for AD, is associated with dysregulation of cholesterol transport (17). Furthermore, metabolite levels and gene expression associated with cholesterol biosynthesis are significantly reduced in brain regions vulnerable to AD pathology (18). However, the exact role of brain cholesterol in AD pathogenesis remains unclear. For example, discrepancies exist regarding neuronal membrane cholesterol content in AD. Both increased and decreased levels of neuronal PM cholesterol have been reported in different studies and models (19, 20, 21, 22).

Cholesterol detection and quantification techniques are critical for understanding its biological functions (23). Traditional methods, such as nuclear magnetic resonance (NMR) Spectroscopy, suffer from low spatial resolution (24), while fluorescent cholesterol analogs and Filipin III staining are limited by nonspecific binding and poor tissue penetration (25). Perfringolysin O (PFO) is a cholesterol-dependent cytolysin produced by the bacterium *Clostridium perfringens*. PFO binds specifically to cholesterol in the cell membrane, triggering a conformational change that results in the formation of pores and subsequent cell lysis (26, 27). Notably, the 13 kDa domain 4 (D4) of PFO is sufficient to associate with cholesterol in PMs when cholesterol concentration exceeds 30–35 mol %. D4 and D4H (D434S mutation-containing) sensors have been widely used to study cholesterol dynamics in *vitro*. However, unlike the purified D4/D4H peptides, the cytosolically-expressed D4/D4H sensors cannot always detect PM cholesterol efficiently, because cholesterol concentrations are variable in different cell types. In addition, the aggregation properties of D4/D4H sensors in cytosol severely impair their membrane targeting (28). To date, several new D4 mutant derivates with significantly lower cholesterol-binding thresholds have been identified, such as YDA (D434A, D434/A463W, Y415A/D434W/A463W) and YDQA (Y415A/Q433W/D434W/A463W), although these remain less validated in living systems (29).

In this study, we further developed the YDQA sensor using the optimized self-associating split-fluorescent protein tags sfCherry3C_1-10_ and sfCherry2_11x3_. The new sensor comprises MyrPalm-sfCherry3C_1-10_ for PM anchoring and sfCherry2_11x3_-YDQA for binding to PM cholesterol. The PM cholesterol-dependent complementation of sfCherry induces fluorescence signals that can be easily detected and quantified. We showcased that the new sensor (named sfPMcho) specifically probes PM cholesterol in cell models and exhibits a significantly higher signal-to-noise ratio than Filipin III. Moreover, we successfully applied the sfPMcho sensor in *C. elegans* nervous system and observed the real-time motility of PM cholesterol. Finally, we delivered the sfPMcho sensor in mouse brains via recombinant adeno-associated viruses (AAVs) and uncovered the PM cholesterol abnormalities associated with AD-related pathology.

## Materials and methods

### Antibodies and chemicals

**Fig. 1:**
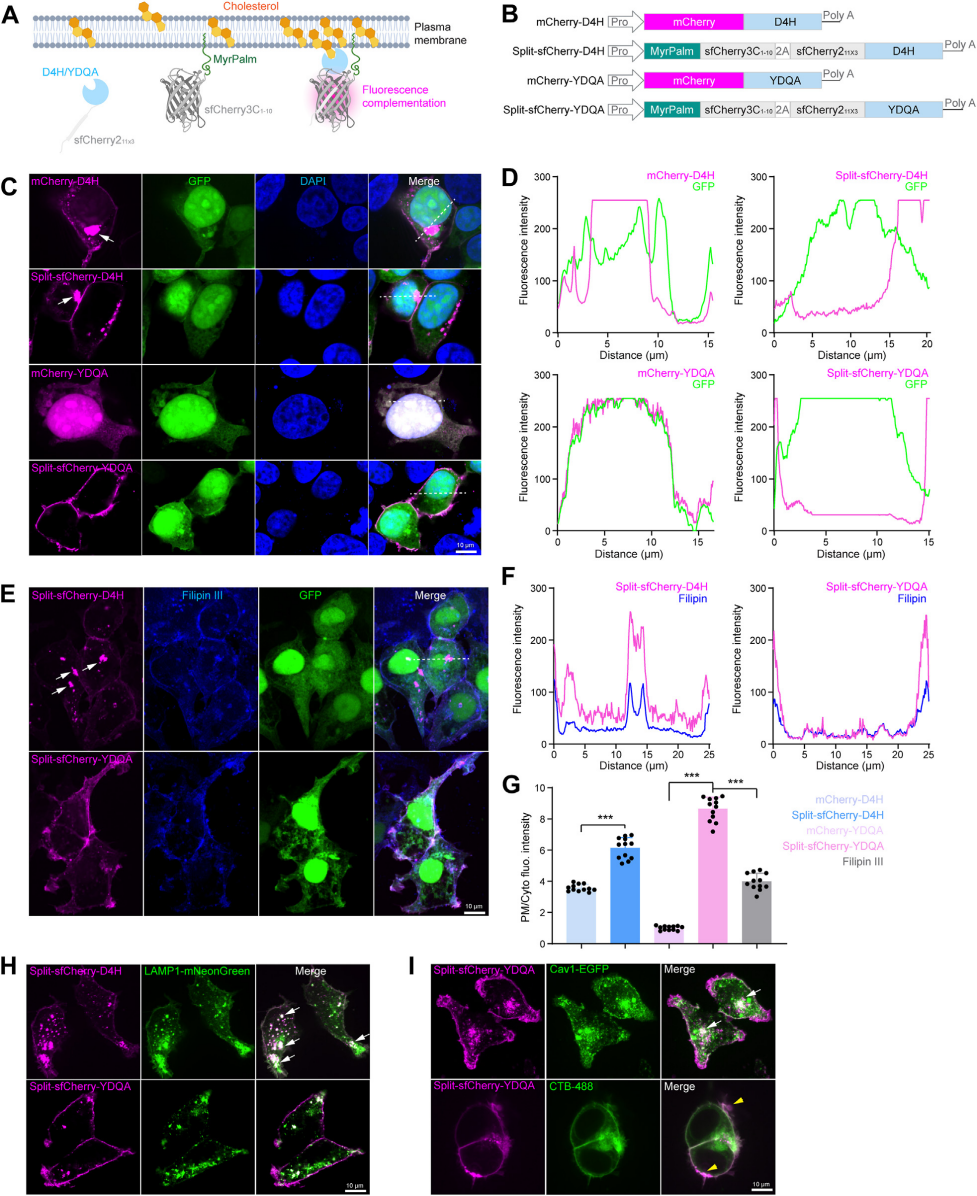
Generation of split-sfCherry-YDQA sensor. A: Schematic design of D4H/YDQA sensors for detecting PM cholesterol. B: Schematic representation of full-length and split-fluorescent protein-tagged D4H/YDQA constructs. C: Co-expression of mCherry-D4H/YDQA or split-sfCherry-D4H/YDQA with GFP in 293T cells. The white arrow indicates aggregates. D: Quantification of membrane and subcellular localization of different cholesterol sensors. E: Cholesterol co-labeling using split-sfCherry-D4H/YDQA and Filipin III in 293T cells. F: Quantification of membrane and subcellular localization of split-sfCherry-D4H/YDQA and Filipin III. G: Quantification of PM/cytosol fluorescence intensity for mCherry-D4H/YDQA, split-sfCherry-D4H/YDQA, and Filipin III. H: Co-expression of split-sfCherry-D4H/YDQA with the lysosomal marker LAMP1-mNeonGreen in 293T cells. I: Cholesterol co-labeling with split-sfCherry-YDQA and lipid raft markers Cav1-EGFP and CTB488. Scale bar: 10 μm.

Antibodies, chemicals, and other reagents used in this study are listed in Supplemental Table 1. The methods and results of antibody validation were provided in Supplementary material and Supplementa Fig. 1.

### Molecular biology

For mammalian expression, cDNAs of D4H, YDQA, sfCherry3c_1-10_ and sfCherry2_11x3_ were synthesized by Synbiob Ltd, China. The plasmids P_cmv_D4H-mCherry, P_cmv_YDQA-mCherry, P_cmv_MyrPalm-sfCherry3c_1-10_-2A-sfCherry2_11x3_-D4H, P_cmv_MyrPalm-sfCherry3c_1-10_-2A-sfCherry2_11x3_-YDQA, P_cmv_MyrPalm-sfCherry3c_1-10_-2A-sfCherry2_11x3_ and P_cmv_MyrPalm-sfCherry3c_1-10_-2A-sfCherry2_11x3_-YDQA^mut^ were constructed using Gibson Assembly (30, 31) and inverse PCR (iPCR)-based mutagenesis (32). All final constructs were verified by Sanger sequencing.

For *C. elegans* expression, cDNAs of YDQA, wrmScarlet_1-10_, wrmScarlet_11x3,_ and DHCR7 were synthesized by Synbiob Ltd, China. The plasmids P_snt-1_::Myr-wrmScarlet_1-10_, P_rgef-1_::wrmScarlet_11x3_-YDQA, P_rgef-1_::wrmScarlet_11x3,_ and P_rgef-1_::DHCR7 were constructed using Gibson Assembly, iPCR-based mutagenesis and Gateway LR Cloning (33, 34). All final constructs were verified by Sanger sequencing.

The detailed maps, cloning procedures, and sequencing data of the above-mentioned plasmids are attached in Supplementary File 1. All the homemade plasmids are free to share when requested for academic research.

For the generation of recombinant adeno-associated viruses (rAAVs), the backbone of pAAV-hSyn-mCherry-3xFLAG-WPRE was generated by double digests at the restriction site of BamHI and HindIII to remove mCherry-3xFLAG. The cDNA of MyrPalm-sfCherry3c_1-10_-2A-sfCherry2_11x3_-YDQA was amplified and subcloned into the backbone using Gibson Assembly. The final construct pAAV-hSyn-MyrPalm-sfCherry3c_1-10_-2A-sfCherry2_11x3_-YDQA-WPRE were in vitro packaged to rAAV (serotype: AAV2/PHP.eB) by Obio Ltd, China. The titer of rAAV was determined by standard qPCR technology (35).

The following plasmids, including pLAMP1-mNeonGreen (#98882), pCav1-EGFP (#27704), pLCK-mTurquoise2 (#98822), pER-mNeonGreen (#137804) and *P*_*myo-3*_*::GFP* (#86713) were purchased from Addgene.

### Cell culture and transfection

293T cells (CSTR:19,375.09.3101HUMSCSP502, gift from Dr Yan Jiang, Fudan University), HeLa Cells (CSTR:19,375.09.3101HUMSCSP504), N2a cells (CSTR:19,375.09.3101MOUSCSP5035) and HT22 cells (CSTR:19,375.09.3101MOUSCSP5419) were originated from National Collection of Authenticated Cell Cultures, China. All the cells were thawed from the frozen stocks (liquid N2) and cultured in standard medium (DMEM supplemented with 10% v/v FBS) at 37°C and 5% CO2. To ensure the cells remain in good condition, the passage number of all the cells was kept below 20, and experiments were conducted between passage numbers 5 and 15. For live-cell imaging experiments, cells were seeded onto 35-mm glass-bottom dishes (coated with Poly-D-lysine, PDL). For fixed-cell immunofluorescence, cells were seeded onto 14-mm microscope cover glass (coated with PDL) in 24-well cell culture plates. Plasmid transfection was performed using the EZ Trans cell transfection reagent.

### Filipin III staining

The Filipin III stock solution was prepared in DMSO (50 mg/ml). For staining of cultured cells, cells grown on coverslips were fixed with 4% paraformaldehyde for 15 min at room temperature. Fixed cells were rinsed three times with PBS and incubated with glycine buffer (1.5 mg/ml in PBS) for 10 min to quench the paraformaldehyde. The cells were then stained with freshly prepared Filipin III working solution (50 μg/ml in PBS) for 1 h at room temperature. For the fixed brain samples, the slices were cryostat-sectioned at 20 μm and incubated with freshly prepared Filipin III working solution for 1 h at room temperature. All Filipin III-stained samples were imaged with the SpinFV-COMD super-resolution confocal microscope using a 405 nm laser.

### MβCD stimulation and live cell imaging

For time-lapse imaging experiments with MβCD stimulation, the cell culture medium was replaced with a 10 mM MβCD-containing medium. After selecting the appropriate laser channels (405 nm, 488 nm, or 561 nm) at suitable powers, a drop of lens oil was added to the 100× NA of the objective of the SpinFV-COMD super-resolution confocal microscope. Next, the sample dish was placed on the microscope, fixed with the matching clamp, and the heater provided by the manufacturer was turned on to maintain the sample at ambient temperature (37°C). The focus was then adjusted to obtain clear images of the cells, and the appropriate cell area was selected. Finally, the autofocus was turned on, and the film recording time was set to 20 min and 20 cycles.

### CTB-488 labeling

The working solution of CTB-488 was freshly prepared in serum-free DMEM at a concentration of 1 μg/ml. The cultured cells were replaced with CTB-488 solution and incubated for 2 h at 37°C. Live-cell imaging was performed as described above.

### *C. elegans* strains and genetics

*C. elegans* strains were maintained using standard methods and cultured at 20°C.

To generate *ycyEx46 [snt-1p::Myr::wrmScarlet*_*1-10*_*; rgef-1p::wrmScarlet*_*11×3*_*; rab-3p::GFP]*, *P*_*snt-1*_*Myr-wrmScarlet*_*1-10*_ (25 ng/μl), *P*_*rgef-1*_*-wrmScarlet*_*11x3*_ (25 ng/μl), *P*_*rab-3*_*::GFP* (15 ng/μl) and *pBluescript* (60 ng/μl) were injected into N2 animals.

To generate *ycyEx52 [snt-1p::Myr::wrmScarlet*_*1-10*_*; rgef-1p::wrmScarlet*_*11×3*_*::YDQA; rab-3p::GFP]*, *P*_*snt-1*_*Myr-wrmScarlet*_*1-10*_ (25 ng/μl), *P*_*rgef-1*_*-wrmScarlet*_*11x3*_*-YDQA* (25 ng/μl), *P*_*rab-3*_*::GFP* (15 ng/μl) and *pBluescript* (35 ng/μl) were injected into N2 animals.

The integrated strain *ycyIs21 [snt-1p::Myr::wrmScarlet*_*1-10*_*; rgef-1p::wrmScarlet*_*11×3*_*::YDQA; rab-3p::GFP]* was generated by UV/TMP integration of *ycyEx52*.

To generate *ycyEx55*, *P*_*rgef-1*_*::DHCR7* (100 ng/μl) and *P*_*myo-3*_*::GFP* (10 ng/μl) were injected into ycyIs21 animals.

### Mouse strains

All experimental procedures were approved by the Fudan University Animal Care and Use Committee (No. 20190221-152). Mice were housed on a 12-h light-dark cycle (7:30 AM light ON and 7:30 PM light OFF) with ad libitum access to food and water. 5X FAD mice (B6SJL-Tg(APPSwFlLon, PSEN1M146LL286V)6799Vas/Mmjax, #034840-JAX, Jackson Laboratory) and APOE KO mice (B6.129P2-Apoetm1Unc/J, #002052, Jackson Laboratory) were used in this study (36, 37). Mouse genotyping was performed according to standard procedures outlined on the Jackson Laboratory website.

### Intracranial injection

Eight-week-old APOE knockout (KO) mice and their littermates were anesthetized with 0.5%–2% isoflurane and placed in the stereotaxic apparatus (E07370-005, RWD). The recombinant adeno-associated viruses (AAVs) (AAV2/PHP.eB-hSyn-mCherry-3xFLAG-WPRE and AAV2/PHP.eB-hSyn-MyrPalm-sfCherry3C_1-10_-2A-sfCherry2_11x3_-YDQA-WPRE) were delivered into the two lateral ventricles (ML±1.00, AP-0.70, DV-1.90) by a microinjection pump (R-480 Nanoliter, RWD) at a volume of 150 nl with a flow rate of 1–3 nl/s. The injection pipette was left at the injection site for 10 min after viral delivery. Mice were then returned to their home cages for recovery after the surgery.

### Jugular vein injection

Six-month-old 5X FAD mice and the littermates were injected (100 μl total volume) into the left jugular vein with a dose of 1 × 10^11^ vg/mouse each of the rAAV (AAV2/PHP.eB-hSyn-MyrPalm-sfCherry3C_1-10_-2A-sfCherry2_11x3_-YDQA-WPRE). Mice were anesthetized with isoflurane, and the AAVs were diluted in sterile PBS to achieve the final injection volume.

### Immunostaining

For immunocytochemistry, cells were fixed with 4% paraformaldehyde (PFA), permeabilized with 0.2% Triton X-100 in PBS, and blocked with 5% FBS in PBS. Primary antibodies were diluted and then incubated overnight at 4°C. After washing with PBS, cultures were incubated with secondary antibodies (1:200) at room temperature for 1 h. For immunohistochemistry, mice were anesthetized with sodium pentobarbital and perfused through the aorta with 0.9% NaCl followed by 4% PFA. The brains were collected and sectioned coronally at 30 μm with the Vibratome (Leica). Free-floating slices were then incubated at 4°C overnight with primary and secondary antibodies sequentially. All samples were rinsed in PBS three times and mounted onto slides, dried, and covered with glycerol:TBS (3:1) containing DAPI. Confocal images were acquired using an FV3000 confocal microscope and analyzed with ImageJ (FIJI).

### Quantification and statistical analysis

All statistical tests and sample sizes are specified in the figure legends and specific sections of the Methods where appropriate. Cells were assigned to experimental groups based on experimental conditions (DNA transfection). Animals were randomly assigned to experimental groups depending on genotype. All data analyses were performed using GraphPad Prism 9.5 (GraphPad). *P* < 0.05 were considered statistically significant.

### Ethical approval

All experimental protocols were approved by the Institutional Animal Care and Use Committee of Fudan University, Shanghai Medical College (IACUC Animal Project Number: 20,160,225-046).

## Results

### Generation of split-sfCherry tagged D4H/YDQA sensors

It has been reported that D4 expressed in the cytosol did not consistently detect PM cholesterol in different cell types, due to PM cholesterol concentration and/or surroundings (eg, phospholipids). Artificial point mutations were introduced in D4 previously to lower its high threshold (>30 mol%) for cholesterol-binding. Here, we tested two types of D4 mutants, D4H (carrying D434S) and YDQA (carrying Y415A/Q433W/D434W/A463W) with cholesterol-binding thresholds at 20 mol% and 1 mol% respectively. The self-associating split-fluorescent protein system is a powerful tool for target labeling and live-cell imaging. The red-colored split-sfCherry has been well-optimized for much-enhanced complementation efficiency and fluorescence signal intensity (38). Here, we designed a cholesterol-dependent split-sfCherry complementation for PM cholesterol detection, as illustrated in Fig. 1A. The sfCherry3C_1-10_ fragment was fused with the MyrPalm tag to facilitate PM targeting. The small fragment sfChery2_11x3_ was fused to D4H or YDQA for cholesterol binding. It has been elucidated that split-sfCherry undergoes a dynamic association/dissociation equilibrium before irreversible complementation and produces substantially low fluorescence (39). Thus, the assisted complementation induced by cholesterol-binding D4H or YDQA evokes bright fluorescence in PM. We generated the constructs split-sfCherry-D4H/YDQA, distinguishing them from the full-length mCherry fused-D4H/YDQA (Fig. 1B). When tested in the cell model (Fig. 1C), mCherry-D4H formed very large aggregates (white arrow) in the cytosol, although it was partially associated with PMs. The aggregates were somehow reduced but not abolished in split-sfCherry-D4H. mCherry-YDQA did not localize to the PM or intracellular aggregation but rather was ubiquitously distributed similar to GFP. Surprisingly, split-sfCherry-YDQA strongly reduced cytosolic signals and predominantly localized on PM distribution (Fig. 1D), indicating its high efficiency in cholesterol-binding.

Filipin III is a widely used cholesterol dye but its use is limited by its weak signal and fast photo-bleaching properties. For comparison, 293T cells were transfected with split-sfCherry-D4H/YDQA and stained by Filipin III subsequently. As shown in Fig. 1E, their signals in PMs evoked by cholesterol showed significant overlap. However, split-sfCherry-YDQA exhibited a significantly higher PM/Cyto fluorescence intensity ratio (Fig 1F, G). The lysosome is a key organelle involved in protein degradation. Co-expressing split-sfCherry-D4H with lysosome marker, LAMP1-mNeonGreen, resulted in many coincident signals in aggregates (white arrow). In contrast, this phenomenon was very rare in the split-sfCherry-YDQA context (Fig. 1H), further suggesting its solubility and PM cholesterol-binding efficiency.

Cholesterol is not evenly distributed in PM. We observed the dense signals at some parts of the membrane when expressing split-sfCherry-YDQA. To further analyze the correlation between cholesterol contents and sensor signals, we examined lipid rafts, the cholesterol-enriched microdomain in PM. We co-expressed split-sfCherry-YDQA with lipid raft marker Cav1-EGFP. Previous studies have shown that the expression of caveolin-1 (Cav1) increases the amount of cholesterol recovered in the lipid rafts (40). In line with this, split-sfCherry-YDQA showed significant overlap with Cav1, suggesting a high abundance of cholesterol, suggesting a high abundance of cholesterol. Alternatively, we tracked lipid raft with the dye CTB-488 and observed a similar signal distribution pattern from split-sfCherry-YDQA (Fig. 1I). These results show that tagging with the split-fluorescent protein overcomes the sensitivity and solubility issues of D4H/YDQA. The split-sfCherry-YDQA represents an optimized sensor that can be used to track PM cholesterol in live cells.

### Split-sfCherry-YDQA sensor detects PM cholesterol dynamics

**Fig. 2:**
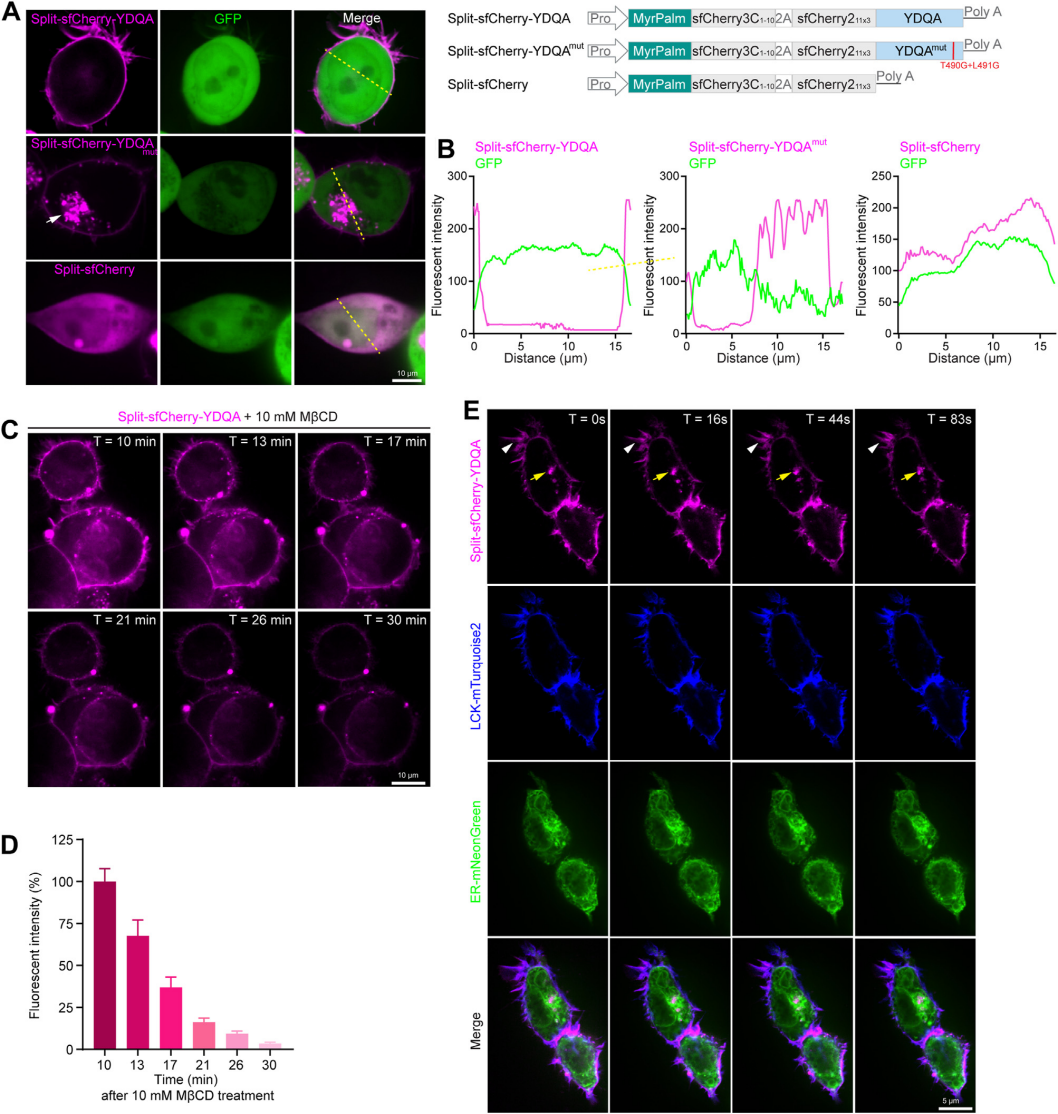
Split-sfCherry-YDQA sensor detects PM cholesterol dynamics. A: Mutagenesis of split-sfCherry-YDQA and their signal pattens when expressed 293T cells. B: Quantification of membrane and subcellular localization of the modified cholesterol sensors. C: Cholesterol depletion assay. PM cholesterol was visualized using the split-sfCherry-YDQA sensor and depleted by treatment with 10 mM MβCD. D: Quantification of fluorescence intensity at the indicated time points. E: Live cell imaging of dynamic cholesterol changes tracked by the split-sfCherry-YDQA sensor. The cell membrane was labeled with LCK-mTurquoise2, and the endoplasmic reticulum (ER) was labeled with ER-mNeonGreen. White arrow: cell membrane. Yellow arrow: ER. Scale bar: 10 μm.

A question to consider is whether the split-sfCherry-YDQA, while providing high detection sensitivity, also induces nonspecific or false-positive signals. This could be due to two reasons: 1. MyrPalm-sfCherry3C_1-10_ stably anchored to PM and recruits too many sfChery2_11x3_ fragments; 2. YDQA carries four artificial point mutations that raise the cholesterol-binding threshold, but may also produce non-specific affinity. As verification, we manipulated the sensor via site-directed mutagenesis. Based on previous studies, the threonine–leucine pair (T490 and L491) is essential for the cholesterol recognition of D4 and the double mutants (T490G + L491G) can abolish its cholesterol-binding without affecting the overall structure (41). We introduced the double mutants to split-sfCherry-YDQA and termed it as split-sfCherry-YDQA^mut^. Compared with the unmutated sensor, we found split-sfCherry-YDQAmut had a very weak PM signal but formed large intracellular aggregates. We further removed the YDQA fragment completely and observed the diffused RFP signals in the cells (Fig 2A, B). As negative controls, these data indicate that split-sfCherry-YDQA has a high specificity in PM cholesterol-binding.

We then focused on split-sfCherry-YDQA and tested its utility in monitoring cholesterol dynamics. It has been established that methyl-β-cyclodextrin (MβCD) selectively extracts cholesterol from PM. We conducted the PM cholesterol depletion assay. Split-sfCherry-YDQA transfected cells were treated with 10 mM MβCD, and image sequences were captured with a time-lapse imaging system starting initiated 10 min post-treatment. As shown in Fig 2C, D, the intense sensor signals in PM decreased gradually along with the drug treatment, reflecting the cholesterol efflux in real-time.

In addition, we found that split-sfCherry-YDQA often detects some small and highly deformed intracellular signals. We speculate that the sensor may track certain organelles with high cholesterol levels stochastically, for example, the Endoplasmic reticulum (ER). ER plays a critical role in cholesterol homeostasis. ER is the main site of cholesterol synthesis and mediates multiple cholesterol transport routes such as PM-ER, ER-Golgi, and Peroxisome-ER. Thus, cholesterol in the ER is in highly abundant and dynamic. We co-expressed ER marker ER-mNeonGreen and PM marker LCK-mTurquoise2 with split-sfCherry-YDQA and performed the short-time live imaging. As expected, the sensor exhibited primary co-localization with LCK-mTurquoise2 (white arrowhead) and a few intracellular puncta (Fig. 2C). Notably, the puncta were localized to ER structures and deformed continuously (yellow arrow), implying the dynamic changes of local cholesterol. Together, these experiments further demonstrate the sensitivity of split-sfCherry-YDQA sensor in tracking PM cholesterol dynamics.

### Application of sfPMcho sensor in *C. elegans* nervous system

**Fig. 3:**
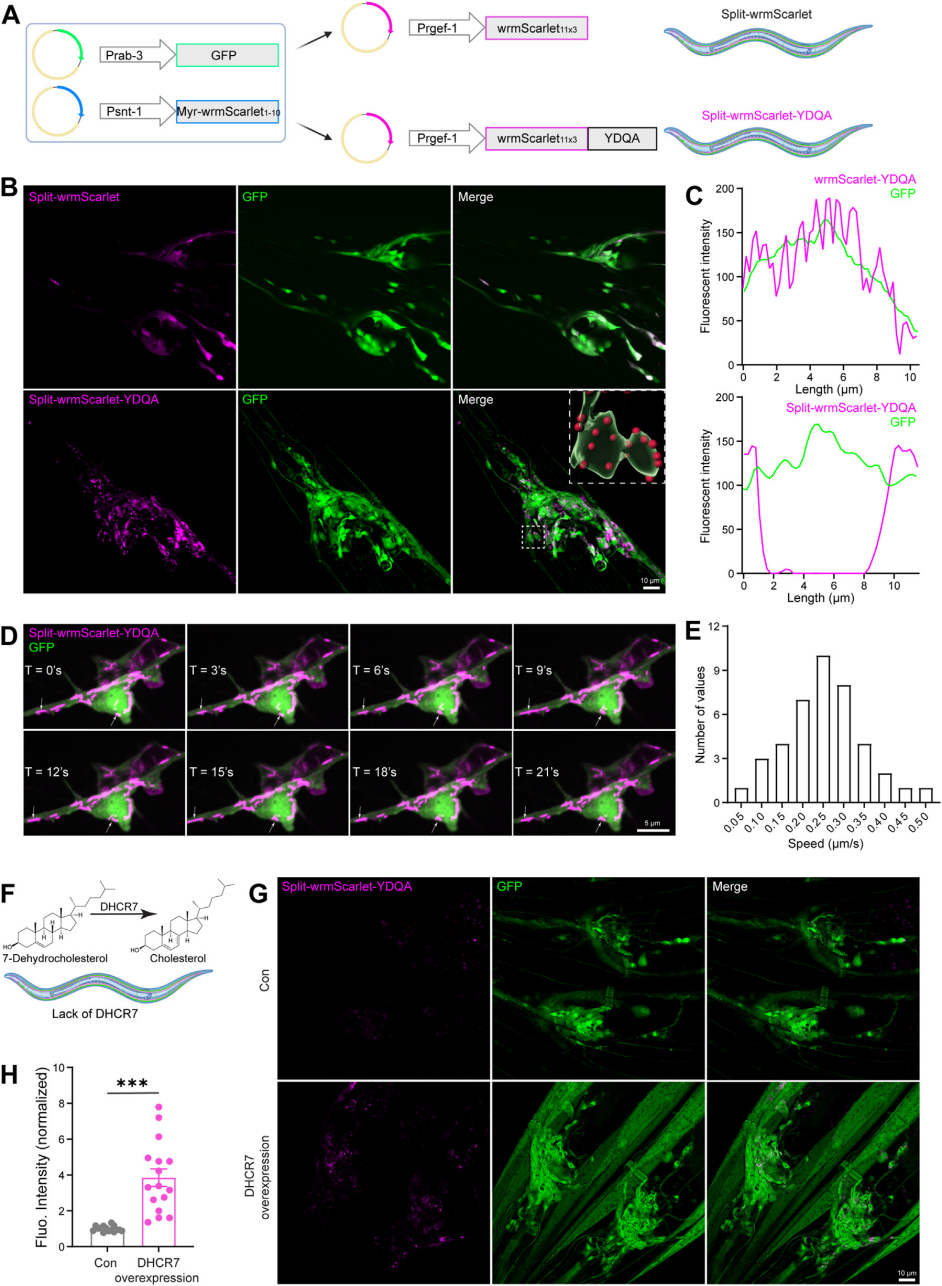
Neuronal PM cholesterol dynamics detected by split-wrmScarlet-YDQA. A: The design of split-wrmScarlet-YDQA. B: Detection of *C. elegans* neuronal PM cholesterol cholesterol using split-wrmScarlet-YDQA. GFP was used to label neurons. C: Quantification of membrane and subcellular localization of wrmScarlet-YDQA and split-wrmScarlet-YDQA. D: Snapshots of neuronal PM cholesterol dynamics visualized by the split-wrmScarlet-YDQA sensor. E: Frequency distribution of PM cholesterol transport speeds. F*: C. elegans* lack the enzyme DHCR7, which converts 7-dehydrocholesterol to cholesterol. G: The split-wrmScarlet-YDQA sensor detects cholesterol increase induced by DHCR7 overexpression. H: Quantification of split-wrmScarlet-YDQA sensor signals reflecting neuronal PM cholesterol content. Scale bar: 10 μm.

To pursue the in *vivo* application of the split-sfCherry-YDQA sensor (hereafter referred to as sfPMcho), we deployed *C. elegans*, a type of eukaryotic, multi-organ, transparent animal with a simple but relatively intact nervous system, as the bridge. To achieve better expression in *C. elegans*, we used the optimized wrmScarlet to replace sfCherry (Fig. 3A). Like the split-sfCherry mutant in cell models (Fig. 2A), split-wrmScarlet (with Myr tag) expressed in *C. elegans* neurons also did not exhibit PM targeting. In contrast, split-wrmScarlet-YDQA showed specific puncta signals in PM, as illustrated by 3D reconstruction (Fig 3B, C).

We further analyzed the PM cholesterol dynamics using the split-wrmScarlet-YDQA sensor. We performed short-time live imaging of the PVD neuron, a type of sensory neuron located in the middle of the body in *C. elegans*. Interestingly, we found that the split-wrmScarlet-YDQA labeled PM cholesterol displayed punctate or segmented structures. These cholesterol-rich fractions are highly dynamic, undergoing constant transportation, fusion, and fragmentation (Fig. 3D). We quantified their motility and revealed the median velocity distribution was approximately 0.2–0.3 μm/s (Fig. 3E).

Next, we generated the transgenic *C. elegans* stably expressing split-wrmScarlet-YDQA in the nervous system. *C. elegans* cannot synthesize cholesterol and must ingest it through the diet. Moreover, *C. elegans* grown in laboratory stigmasterol-supplemented media utilize 7-dehydrocholesterol rather than cholesterol as the predominant sterol type (51%) (42). 7-Dehydrocholesterol is a precursor in the cholesterol synthesis pathway that is catalyzed by DHCR7 (7-Dehydrocholesterol reductase) (Fig. 3F). DHCR7 is essential for cholesterol homeostasis in mammals. Mutations that reduce the catalytic efficiency or even ablate DHCR7 lead to significant pathology in humans, known as Smith-Lemli-Opitz syndrome (SLOS) (43). Interestingly, *C. elegans* normally lacks DHCR7 but exhibits no such pathology. Previous studies have shown that transgenic expressing human DHCR7 can produce significantly more cholesterol (∼80%) in *C. elegans* (44). As a validation, we overexpressed human DHCR7 in those split-wrmScarlet-YDQA transgenic worms. As a result, the manipulation dramatically enhanced the split-wrmScarlet-YDQA signal, reflecting the increased cholesterol concentration (Fig 3G, H). These data confirm that the sfPMcho sensor detects neuronal PM cholesterol dynamics sensitively in vivo.

### Visualize neuronal PM cholesterol abnormalities in AD-related pathology

**Fig. 4:**
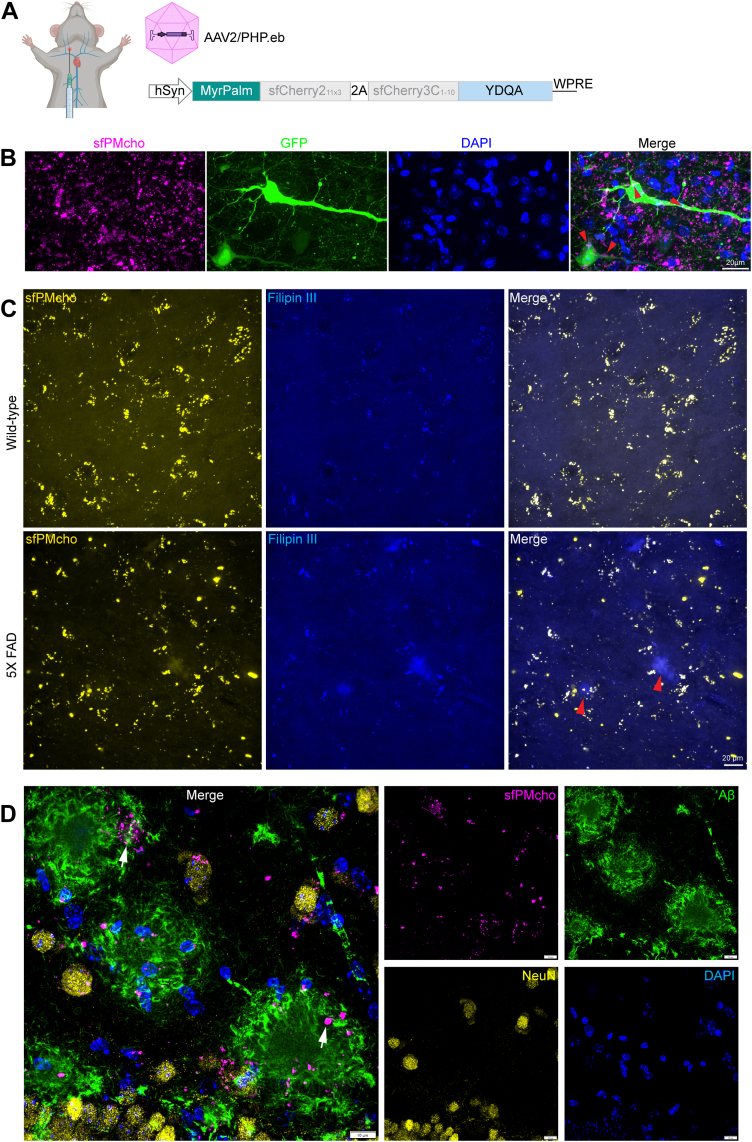
Visualization of neuronal PM cholesterol abnormalities in 5X FAD mice. A: Schematic representation of the virus injected into the jugular vein. B: A representative image of PM cholesterol tracked by the sfPMcho sensor. Red arrow: PM cholesterol in neuronal cell bodies and neurites. C: Co-labeling of PM cholesterol using the sfPMcho sensor and Filipin III in cortical regions. Red arrow: amyloid plaques. Scale bar: 100 μm. D: Co-localization analysis of PM cholesterol (visualized by the sfPMcho sensor), amyloid plaques (Aβ), and neuronal cell bodies (NeuN). Scale bar: 10 μm.

To apply sfPMcho sensor in the brain, we packed the sensor into AAV-PHP.eB, a versatile tool that crosses the blood–brain barrier after intravenous administration and broadly transduces cells throughout the brain (Fig. 4A). Using this strategy, we delivered the sfPMcho sensor into 5X FAD AD model mice and their wild-type littermates. As shown in Fig. 4B, sfPMcho sensor was expressed in neurons and evokes very dense fluorescence signals in the brain. When aligned with individual neurons, we found that the punctate signals were located in the PMs of the neuronal body and projections, similar to the distribution pattern seen in *C. elegans*.

Previously, the visualization of brain cholesterol has predominantly been achieved through Filipin III staining. To verify the efficiency of the sfPMcho sensor in the brain, we performed Filipin III staining simultaneously. In mouse cortical regions (Fig. 4C), PM cholesterol detected by Filipin III and sfPMcho sensor were both displayed as puncta and their signals were almost fully overlapped. However, the signals of Filipin III were faintly visible, and the signal-to-noise ratio was very low. We observed that Filipin III could directly label amyloid plaque-like structures in the brains of 5X FAD mice (red arrowheads), while the sfPMcho sensor primarily tracked cholesterol scattered around the plaque. This difference demonstrates the specificity of the sfPMcho sensor because it was expressed in neurons. Although senile plaques are cholesterol-enriched, they consist mainly of deposited granules and remnant neuronal protrusions. To verify this, we immunostained amyloid plaque with Aβ and NeuN antibodies (45, 46). Clearly, cholesterol detected by sfPMcho sensor was predominantly distributed around the periphery of amyloid plaques rather than in the core (Fig. 4D), presumably originating from nearby neural projections or debris. These results demonstrate that the sfPMcho sensor is sensitive and stable in vertebrate brains.

**Fig. 5:**
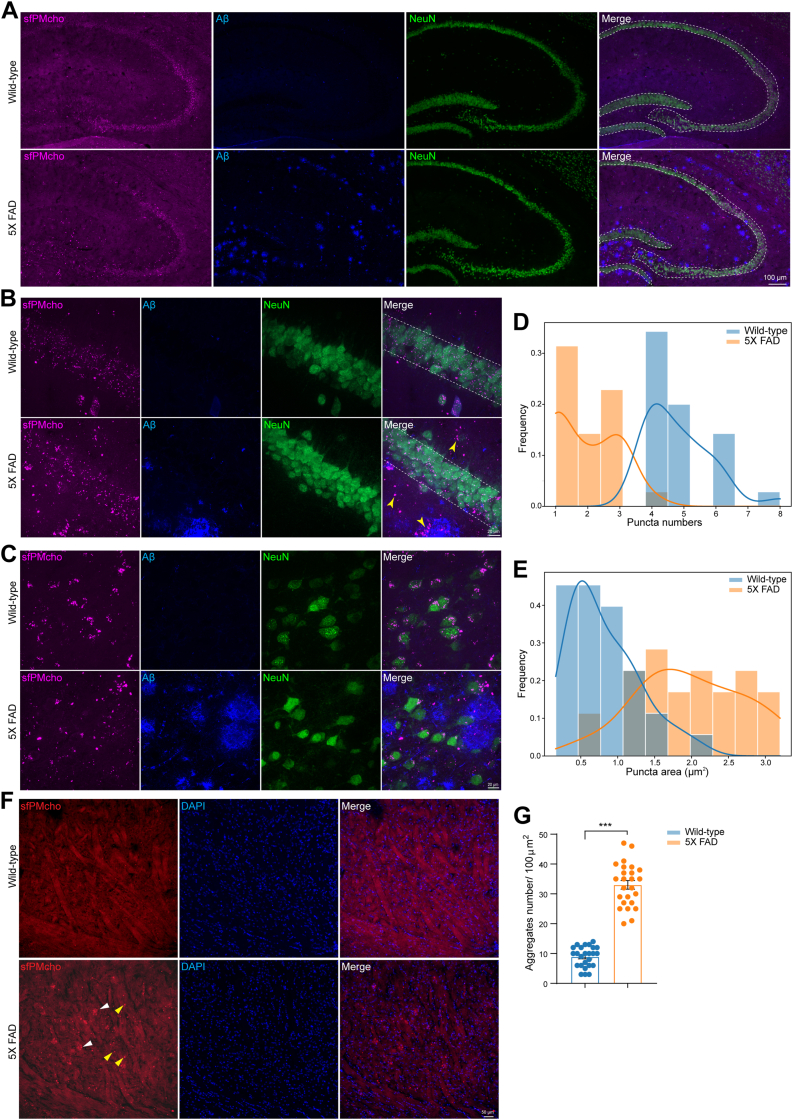
PM cholesterol abnormalities in neuronal cell bodies and nerve fibers. A: Neuronal PM cholesterol was visualized using the sfPMcho sensor in hippocampal regions. Amyloid plaques were stained with an Aβ antibody. Neurons were labeled with a NeuN antibody. Scale bar: 100 μm. B: Neuronal PM cholesterol changes in CA1 regions between Wild-type and 5X FAD mice. Scale bar: 20μm. C: Neuronal PM cholesterol changes in cortex regions between Wild-type and 5X FAD mice. Scale bar: 20μm. D: Frequency distribution and linear fitting plots of sensor signals (puncta numbers) in Wild-type and 5X FAD mice. E: Frequency distribution and linear fitting plots of sensor signals (puncta area) in Wild-type and 5X FAD mice. F: PM cholesterol accumulation in nerve fibers of 5X FAD mice. White arrow: amyloid plaques. Yellow arrow: locally aggregated cholesterol. Scale bar: 50μm. G: Quantification of aggregated cholesterol in nerve fibers of wild-type and 5X FAD mice.

Then, we investigated PM cholesterol changes in the hippocampus, which is a brain region crucial for learning and memory. In wild-type mice, the sfPMcho sensor signals were predominantly localized to NeuN-labeled neuronal bodies. However, many large puncta were distributed outside of neurons and scattered around amyloid plaques in 5X FAD mice (Fig. 5A). Carefully quantifying CA1 neurons at high magnification revealed abnormalities in cholesterol distribution. PM cholesterol tracked by sfPMcho sensor in wild-type neurons was displayed as small and dense puncta, whereas in neurons from 5X FAD mice, the puncta became large and sparse (Figs. 5B, D and E). The striking difference was also found in other brain regions, such as the cortex (Fig. 5C). We speculate that those scattered cholesterol signals outside the neuronal bodies in 5X FAD mice may originate from neural projections or cellular debris. Thus, we surveyed white matter, which is the structurally distinct brain region composed of nerve fibers. We found significant cholesterol aggregation and deposition in white matter from 5X FAD mice (Fig 5F, G). Together, these data suggest complex alterations in neural PM cholesterol in AD-related pathology visualized by the sfPMcho sensor.

### APOE KO induces abnormal neuronal PM cholesterol distribution in the brain

**Fig. 6:**
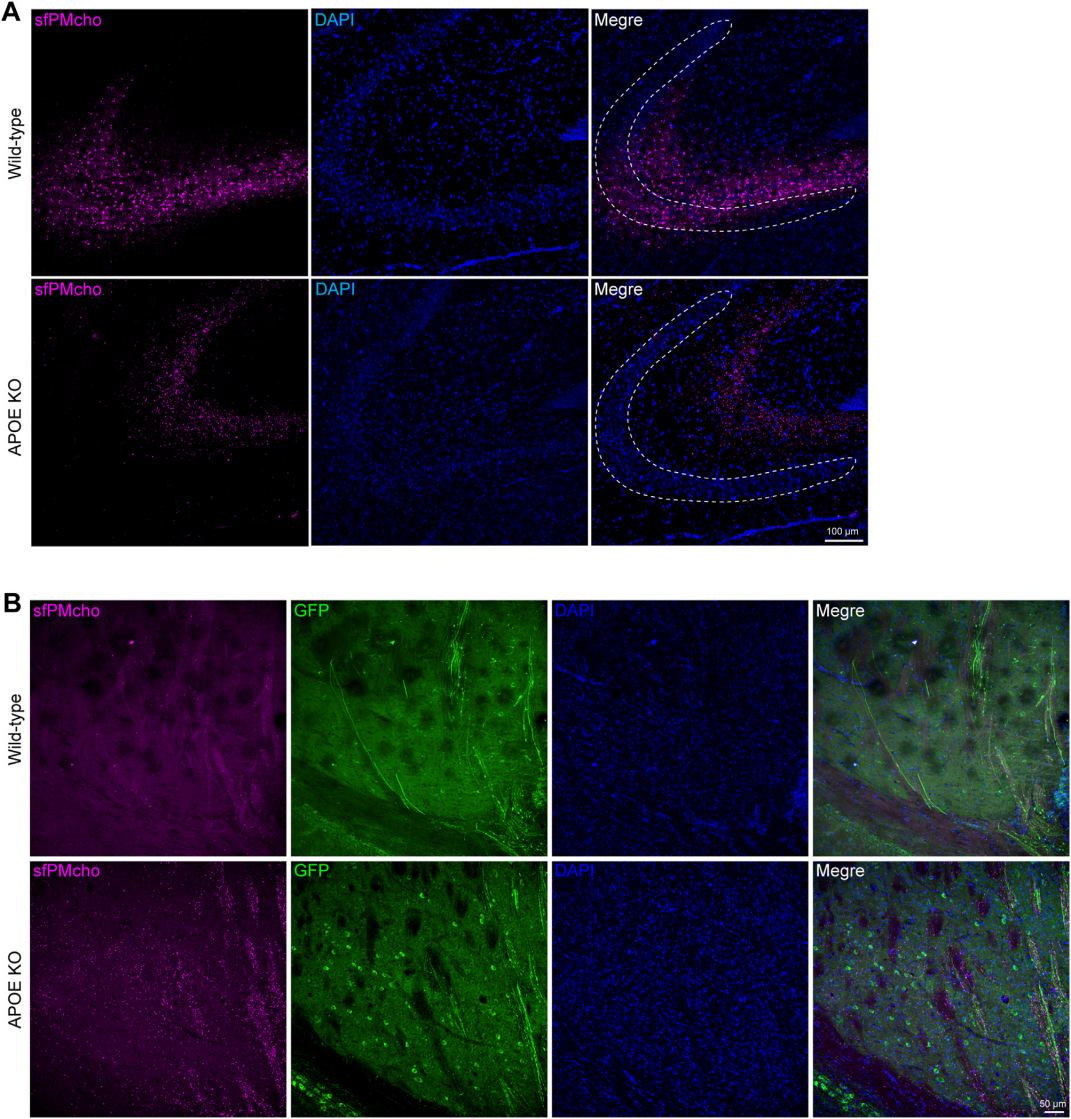
APOE KO induces abnormal neuronal PM cholesterol distribution in the brain. A: PM cholesterol distribution was abnormal in the CA3 region of APOE KO mice. The white dotted area indicates neuronal cell bodies. Scale bar: 100 μm. B: PM cholesterol accumulation in nerve fibers of APOE KO mice. Scale bar: 50μm.

Finally, we explored the influence of APOE, the major genetic risk determinant of AD, on neuronal PM cholesterol in the brain. After stereotaxic injection of the sfPMcho sensor into the lateral ventricles, we observed a striking pattern of neuronal PM cholesterol distribution. As shown in Fig. 6A, in wild-type animals, cholesterol was detected in both CA3 neuron bodies and the proximal parts of neurites. However, in APOE knockout (KO) animals, cholesterol was detected only in the distal part of CA3 neurites. We further examined the striatum and found that APOE deficiency caused significant cholesterol accumulation in nerve fibers (Fig. 6B). A similar phenomenon observed in the 5X FAD mice and APOE KO mice has never been reported. Although we have not yet understood the cause and pathologic implication of this type of cholesterol abnormality, it highlights the value of developing and using new tools in cholesterol research, such as sfPMcho sensor.

## Discussion

Cholesterol plays an important role in the physiology and pathology of the nervous system. In vivo visualization of cholesterol has remained challenging (28, 29, 47, 48). In this study, we generated a new tool, the sfPMcho sensor, for the efficient detection of neuronal PM cholesterol across various models. Using the sfPMcho sensor, we observed an unreported cholesterol abnormality in AD-related pathology.

Although both D4H and YDQA have been reported as genetically encoded sensors for cholesterol detection in vitro, they have significant limitations (Fig. 1C). D4H forms large aggregates (28), while YDQA is broadly distributed throughout the cell, hampering their utility in sensing PM cholesterol. Here, we incorporated the complementary split-fluorescent protein system to enhance their efficiency in detecting PM cholesterol. The split-fluorescent protein has been extensively modified for a wide range of applications including protein labeling and organelle visualization (49). In this system, the 11^th^ β-strand of FP (FP_11_,) is separated from the remainder of FP (FP_1–10_). A specific fluorescence signal is detected when FP_1–10_ reconstitutes with FP_11_ to generate a functional protein. Unlike the classic split-GFP, split-sfCherry undergoes a reversible association-dissociation equilibrium before entering an irreversible process of maturation. Therefore, the overall fluorescent brightness of split-sfCherry is substantially weak (38, 39). In other words, additional assistance enhances the complementation of split-sfCherry and induces significantly stronger signals (39).

Here, we designed a spatial proximity-assisted complementation of split-sfCherry. The sfCherry3C_1-10_ fragment was fused with the MyrPalm, MyrPalm, a membrane-anchoring sequence. The small fragment sfChery2_11x3_ was linked to D4H or YDQA for cholesterol binding. Thus, cholesterol-binding D4H or YDQA-induced assisted complementation results in bright fluorescence at the PM. Additionally, although PFO and its derivatives are known to disrupt cholesterol transport, traditional sensors fused with full-length reporters, such as GFP, may not be suitable for analyzing cholesterol dynamics (50). Given that the detection threshold of YDQA for cholesterol is as low as 1 mol% (51), subtle changes in PM cholesterol can be detected by the split-sfCherry-YDQA sensor signals. We performed a series of tests demonstrating that the split-sfCherry-tagged YDQA sensor is highly sensitive and specific in detecting PM cholesterol and only incidentally detects organelles with high cholesterol content under exceptional circumstances, such as the endoplasmic reticulum (Fig. 2) (52). It exhibits a significantly higher signal-to-noise ratio than Filipin III (53). Notably, we found that split-sfCherry could not effectively alleviate the aggregation of D4H, which is likely due to the inherent properties of D4H.

It is important to note that although cholesterol constitutes roughly 40 mol% of all lipids in PM, the distribution is uneven (54). The D4 sensor has been used to define the “accessible” cholesterol pool, which counts for roughly 10% PM lipids and becomes inaccessible when PM cholesterol falls below 30 mol% or and/or forms complex with other phospholipids and sphingomyelin in local environments (55, 56). The ultralow cholesterol-binding threshold of YDQA (1 mol%) makes it possible to detect those inaccessible cholesterol.

To explore the in vivo application of sfPMcho sensor, we first used *C. elegans* before mice. The advantages of using *C. elegans* are twofold: 1) The neuronal PM cholesterol content in *C. elegans* is extremely low (∼2 mol %), approximately one-tenth of that in mammals (57, 58). This suggests that if the sensor successfully detects PM cholesterol in *C. elegans*, it may be more readily applicable in more complex models. 2) *C. elegans* cannot synthesize cholesterol independently and instead relies on exogenous sources (44, 59). This makes it an ideal model for studying cholesterol dynamics. Using the sfPMcho sensor, we successfully observed and quantified PM cholesterol dynamics in real time (Fig 3C, D). Moreover, we manipulated cholesterol content in *C. elegans* and subsequently monitored it using the sfPMcho sensor. *C. elegans* is a widely used model organism in development, neuroscience, and cell biology. The successful application of the sfPMcho sensor will also facilitate the investigation of other biological processes related to cholesterol homeostasis and function (60, 61, 62).

Cholesterol has been deeply implicated in the pathologies of AD (17, 18, 63, 64). Studies have demonstrated that brain cholesterol levels are closely linked to the development of AD. Most brain cholesterol is localized in myelin sheaths, neuronal membranes, and synaptic structures, all of which are critically affected in AD (65, 66). During AD progression, brain cholesterol metabolism becomes dysregulated, affecting both cholesterol influx and efflux in neurons, which can result in either cholesterol accumulation or depletion. (67, 68, 69). On the one hand, excess cholesterol accumulation enhances Aβ production (53, 70). Cholesterol forms lipid rafts that enhance the activity of β- and γ-secretase (71, 72), thereby accelerating Aβ production and aggregation, and promoting the formation of amyloid plaques (73). Conversely, cholesterol deficiency in the brain can impair neuron survival and synaptic plasticity, as cholesterol is crucial for maintaining membrane fluidity, supporting synapse formation, and facilitating neurotransmission (19, 74, 75). Therefore, cholesterol alterations in AD pathology are highly complex. Using the sfPMcho sensor, we were able to reveal subcellular-level cholesterol abnormalities for the first time. In the 5X FAD mouse model, PM cholesterol became sparse and locally aggregated in neuronal bodies while significantly accumulating in nerve fibers.

APOE is a key apolipoprotein essential for cholesterol transport and clearance in the brain. There are three common polymorphisms in the APOE gene (APOE ε2, ε3, and ε4), with APOE ε4 being the major genetic risk factor for AD (76). The APOE ε4 allele is thought to represent a putative loss-of-function variant and may be associated with an imbalance in cholesterol metabolism and impaired Aβ clearance (77). In APOE knockout (KO) mice, we observed a significant alteration in the distribution of cholesterol within neurons. PM cholesterol levels decreased markedly in neuronal cell bodies and the proximal parts of neurites while increasing significantly in distal neurites. Additionally, cholesterol accumulated in large amounts in nerve fibers, resembling the pattern observed in 5X FAD mice. Taken together, APOE dysfunction resulted in significant cholesterol abnormalities. The phenotype of cholesterol accumulation in nerve fibers closely resembled that observed in the AD model, which may provide insights into the mechanisms of AD induced by APOE ε4.

Imperative utilization of biosensors has acquired paramount importance in the field of biological and medical science. In cooperating with the advanced optical imaging and quantification technology, the genetically encoded fluorescence sensors such as redox (pHaROS, NADP-Snifit) (78, 79), neurotransmitters (iGluSnFR, GRAB-DA) (80, 81), and Kinases/Phosphatases (AMPfret, Rluc-PTEN-YFP) (82, 83) paved way to understand those biological process both in vitro or in vivo. We think our pioneering work, the sfPMcho sensor, raises a valuable strategy for the further development of cholesterol biosensors. Considering the ultralow cholesterol-binding threshold of YDQA, it is also possible to be tagged with different optical elements (e.g., NanoLuc) and better quantified (e.g., Bioluminescence resonance energy transfer), which we have used in D4 (84). In addition, not only D4 or YDQA but also other cholesterol-sensing/binding molecules such as Ostreolysin A (85), Nakanori (86), GRAM (28), or LYCHOS (87) may be developed as sensors for different purposes and complement each other. More importantly, all these sensors need to be extensively tested and continuously optimized, so open access for the scientific community is a matter of course.

In summary, our study introduces a novel tool for the in vivo detection of neuronal PM cholesterol and showcases its application in investigating cholesterol abnormalities in AD-related pathology. Further development based on this sensor or the above-mentioned strategy is to be expected.

## Data availability

The data supporting the findings of this study are contained within the article and the supplementary information. The reagents and other data are available upon request from the corresponding author Dandan Wang.

## Supplemental data

This article contains supplemental data.

## Conflict of interest

The authors declare that they have no conflicts of interest with the contents of this article.

## Figures and Tables

**Fig S1.tif:**
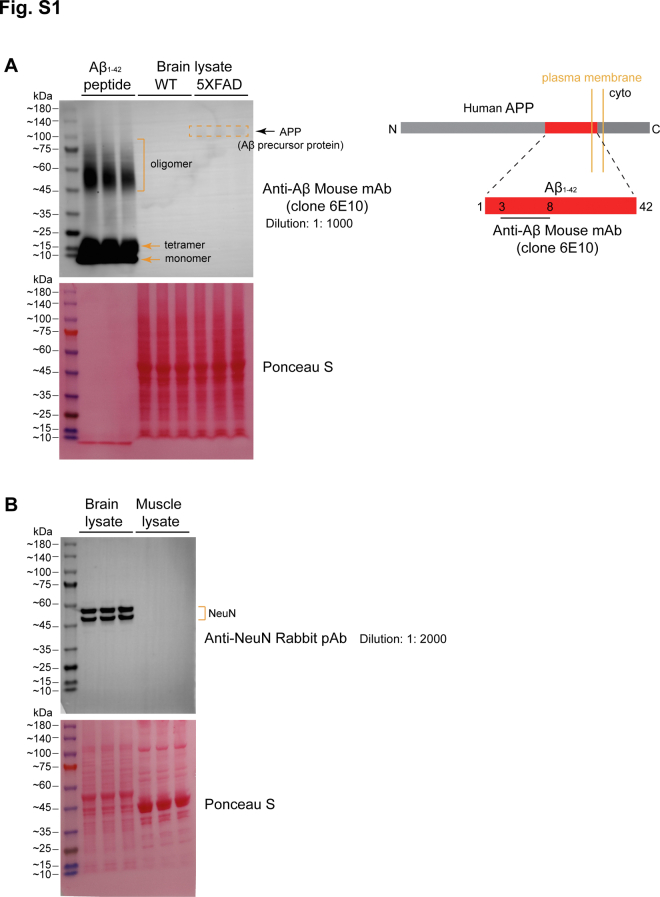


## Supplementary data

pDW129 D4H-mCherry.dna

pDW151 wrmScarlet11x3-YQDA.dna

pDW160 wrmScarlet1-10.dna

pDW169 DHCR7.dna

pDW170 GFP.dna

pDW175 wrmScarlet11x3.dna

pDW274 Split-sfCherry-YDQA.dna

pDW275 Split-sfCherry-D4H.dna

pDW276 YDQA-mCherry.dna

pDW312 Split-sfCherry-YDQAmut.dna

pDW313 Split-sfCherry.dna

pDW315 AAV-sfPMcho.dna

∼$Plasmid information.xlsx

Plasmid information.xlsx

Pdw129-pdw129-f1.ab1

Pdw129-pdw129-f2.ab1

Pdw151.ab1

PDW160-F.ab1

PDW160-R.ab1

PDW160-R1.ab1

1-Prgef1-F.seq

169-169-Primer-R1.ab1

169-169-primer-R2.ab1

170-170-primer-R1.ab1

170-170-primer-R2.ab1

Pdw170.ab1

175-175-primer-R1.ab1

Pdw175.ab1

274-274-primer-R2.ab1

274-274-primer-R3.ab1

Pdw274-f.ab1

Pdw275-f.ab1

Pdw275-r.ab1

276-276-Primer-R1.ab1

276-276-primer-R2.ab1

Pdw312-f.ab1

Pdw312-f2.ab1

Pdw313-f.ab1

Pdw313-f2.ab1

Pdw313.ab1

Pdw315-SEQ2930-Premix-primer.ab1

Pdw315-SEQ2931-Premix-primer.ab1

Pdw315-SEQ2932-Premix-primer.ab1

Supplementary Table 1.docx

Supplementary material.docx
