# Supplementary material for: Visualize neuronal membrane cholesterol with split-fluorescent protein tagged YDQA sensor
Source: J Lipid Res. 2025 Mar 19;66(5):100781. doi: 10.1016/j.jlr.2025.100781 (PMC12147230; doi:10.1016/j.jlr.2025.100781)
Supplement: Supplementary Table 1.docx [file mmc44.docx]

| Reagents | Brand | Catalog |
| --- | --- | --- |
| Anti-NeuN Rabbit pAb  Anti-β-Amyloid (Aβ) Mouse mAb  Goat-anti-Mouse IgG, AF488  Goat-anti-Rabbit IgG, AF647  Goat-anti-Rabbit IgG, AF488  EZ Trans Cell Transfection Reagent  DMEM (Dulbecco's Modified Eagle Medium)  Opti-MEM  PBS buffer  Poly-D-Lysine  0.25% Trypsin-EDTA  FBS (Fetal Bovine Serum)  Methyl-β-cyclodextrin  Filipin III  Paraformaldehyde (PFA)  CTB488  Gibson Assembly® Cloning Kit  Gateway™ LR Clonase™ II Enzyme mix  KOD-Plus- Mutagenesis Kit  BamHI-HF  HindIII-HF | Oasis Biofarm  Biolegend  Oasis Biofarm  Oasis Biofarm  Oasis Biofarm  Life-iLab  Gibco  Gibco  Biosharp  Gibco  Gibco  Gibco  Sigma  MCE  Sinoreagent  Thermo Fisher  NEB  Invitrogen  TOYOBO  NEB  NEB | OB-PRB039-01  SIG-39320  G-MS488  G-MS647  G-RB488  AC04L091  11995065  31985070  BL302A  A3890401  25200-072  10091-148  332615-5G  HY-N6718 G75927B  C34775  E5510S  11791020  SMK-101  R3136S  R3104S |
