# Supplementary material for: Visualize neuronal membrane cholesterol with split-fluorescent protein tagged YDQA sensor
Source: J Lipid Res. 2025 Mar 19;66(5):100781. doi: 10.1016/j.jlr.2025.100781 (PMC12147230; doi:10.1016/j.jlr.2025.100781)
Supplement: Supplementary material.docx [file mmc45.docx]

In this study, we used two antibodies for immunostaining. To verify their specificity in recognizing their targets, we performed immunoblotting in combination with purified peptides and tissue lysates.

Materials and Methods:

Oligomeric Aβ preparation

The purified Aβ_1-42_ peptide was purchased from GL Biochem (China). Considering its aggregation property, we prepared the Aβ oligomers for analysis as described previously(1). Aβ_1-42_ peptide was solubilized in DMSO at a concentration of 5 mM at room temperature. Then, the aliquot was diluted with pre-cold DMEM/F-12 medium ( 11320033, Gibco) to 100 μM. After vertexing for 15 seconds, the solution was transferred to 4°C and incubate for 24 h. The final product, i.e., oligomeric Aβ was stored at 4°C.

Tissue lysates preparation

Six-month-old 5X FAD mice (B6SJL-Tg(APPSwFlLon,PSEN1M146LL286V)6799Vas/Mmjax, #034840-JAX, Jackson Laboratory) and their littermates were anesthetized with isoflurane and dissected to collect their hippocampus and gastrocnemius. Then, the tissues were homogenized in Radio-Immunoprecipitation Assay Buffer (RIPA: 50mM Tris-HCl, pH 7.4, 0.1% SDS, 1% NP-40, 0.25% sodium deoxycholate, 150mM NaCl, 1mM EDTA, 1mM EGTA and protease inhibitors) at 4°C and centrifuged at 15,000×g for 20 minutes to obtain the supernatants. The protein concentrations were determined using the Pierce™ BCA Protein Assay Kit (23225, Thermo) and diluted to 4 μg/μL.

Immunoblotting

Equal amounts of 2X Laemmli SDS Sample Buffer were added to the oligo Aβ solution and tissue lysates, respectively. Then, the samples were heated to 95°C for 5 min. The protein samples (10 μL of each) were separated by SDS-PAGE electrophoresis and transferred to PVDF membranes (1620177, BIO-RAD). The membranes were blocked with 5% skim milk for 1 h at room temperature and then incubated with primary antibodies overnight at 4°C. After three times washing with TBST (0.1% Tween), the membranes were incubated with HRP conjugated secondary antibodies in 5% skim milk for 1h at room temperature. Membranes were washed and signals were developed using ECL Plus western blot substrate. Western blotting images were obtained using KwikQuant Pro Imager (Kindle Biosciences).

Results:

We first verified the mouse monoclonal Aβ antibody (clone: 6E10). Deposition of Aβ in the brain is a pathological hallmark of Alzheimer's disease. There are two major isoforms of Aβ: the 42-residue Aβ_1-42_ and the 40-residue Aβ_1-40_. The only difference between Aβ_1-42_ and Aβ_1-40_ is the two additional C-terminal residues on Aβ_1-42_. However, Aβ_1-42_ is the major component of amyloid plaques in AD brains. As illustrated in Fig. S1, the antigen of this antibody lies within amino acids 3-8 of Aβ_1-42_, the cleaved short peptides from human APP (Aβ precursor protein). This antibody is highly specific to human APP and Aβ. Therefore, it did not detect any proteins from brain lysates of WT mice. In contrast, it recognized the transgenically expressed human APP from brain lysates of 5XFAD mice, although the signal is weak (due to short exposure time, 0.5s). It is important to highlight that although Aβ is produced in large quantities and aggregates to form senile plaques in transgenic mice, it is usually difficult to detect by conventional biochemical extractions and immunoblotting methods, but immunostaining is remarkable. This is true for the vast majority of anti-Aβ antibodies (2). In fact, the antibody (clone: 6E10) has been very successful in detecting purified Aβ (either monomer or oligomer). As a conclusion, the mouse monoclonal Aβ antibody (clone: 6E10) is very specific in detecting human Aβ and suitable for immunostaining.

We then verified the NeuN antibody. NeuN protein is a nuclear protein specifically expressed in neurons in the vertebrate nervous system(3). It is a marker for mature neurons and is used in neuroscience research and diagnostics(4). In this study, we have used a rabbit polyclonal NeuN antibody for indicating neurons in brains. Most of the peripheral organs/tissues contain only nerve fibers without neuronal bodies and nucleus, such as muscles. As a result, the antibody we used in this study detected very specific protein bands in brain lysates, while no signal could be detected in muscle lysates. As a reference, protein bands in brain and muscle lysates also show different patterns under Ponceau S staining. To clarify, it is normal for the NeuN antibody to detect two protein bands that are very similar, as is the case with most anti-NeuN antibodies(5, 6).

**Reference:**

1. Stine, W. B., L. Jungbauer, C. Yu, and M. J. LaDu. 2011. Preparing synthetic Abeta in different aggregation states. *Methods Mol Biol* **670**: 13-32.

2. Youmans, K. L., L. M. Tai, T. Kanekiyo, W. B. Stine, Jr., S. C. Michon, E. Nwabuisi-Heath, A. M. Manelli, Y. Fu, S. Riordan, W. A. Eimer, L. Binder, G. Bu, C. Yu, D. M. Hartley, and M. J. LaDu. 2012. Intraneuronal Abeta detection in 5xFAD mice by a new Abeta-specific antibody. *Mol Neurodegener* **7**: 8.

3. Mullen, R. J., C. R. Buck, and A. M. Smith. 1992. NeuN, a neuronal specific nuclear protein in vertebrates. *Development* **116**: 201-211.

4. Gusel'nikova, V. V., and D. E. Korzhevskiy. 2015. NeuN As a Neuronal Nuclear Antigen and Neuron Differentiation Marker. *Acta Naturae* **7**: 42-47.

5. Cheung, Y. T., W. K. Lau, M. S. Yu, C. S. Lai, S. C. Yeung, K. F. So, and R. C. Chang. 2009. Effects of all-trans-retinoic acid on human SH-SY5Y neuroblastoma as in vitro model in neurotoxicity research. *Neurotoxicology* **30**: 127-135.

6. Sanchez-Bezanilla, S., M. Nilsson, F. R. Walker, and L. K. Ong. 2019. Can We Use 2,3,5-Triphenyltetrazolium Chloride-Stained Brain Slices for Other Purposes? The Application of Western Blotting. *Front Mol Neurosci* **12**: 181.
